# Determination of Trace Antimony (III) in Water Samples with Single Drop Microextraction Using BPHA-[C_4_mim][PF_6_] System Followed by Graphite Furnace Atomic Absorption Spectrometry

**DOI:** 10.1155/2018/8045324

**Published:** 2018-08-01

**Authors:** Xiaoshan Huang, Mingxin Guan, Zhuliangzi Lu, Yiping Hang

**Affiliations:** School of Chemistry and Chemical Engineering, South China University of Technology, Guangzhou 510640, China

## Abstract

A new sensitive method for antimony (III) determination by graphite furnace atomic absorption spectrometry (GFAAS) has been developed by using N-benzoyl-N-phenylhydroxylamine (BPHA) and 1-butyl-3-methylimidazolium hexafluorophosphate ([C_4_mim][PF_6_]) single drop microextraction. The single drop microextraction (SDMM) system is more competitive compared with other traditional extraction methods. Under the optimized conditions, the limit of detection (signal-to-noise ratio is 3) and the enrichment factor of antimony (III) are 0.01 *μ*g·L^−1^ and 112, respectively. The relative standard deviation of the 0.5 *μ*g·L^−1^ antimony (III) is 4.2% (n=6). The proposed method is rather sensitive to determinate trace antimony (III) in water.

## 1. Introduction

Antimony (Sb) compounds have been extensively applied for various industrial materials such as glass, semiconductors, and pharmaceutical products [1–3]. As Sb can be easily exposed to the surface water, it has been considered as a priority pollutant in drinking water in the United States, Canada, Europe, and Japan while their action levels are ranged from 2 to 6 *μ*g·L^−1^ [4]. Sb has two kinds of valence states including Sb (III) and Sb (V), and different chemical forms of them have different levers of toxicity. In inorganic chemistry, the compounds of Sb (III) are almost ten times more poisonous than the compounds of Sb (V) [5–7]. Many diseases including respiratory tract irritation, dermatitis, conjunctivitis, suppuration of the nasal septum, gastritis, or cellular damage in the lungs, heart, and kidneys will be triggered by excessive exposure to Sb (III) [8, 9]. Thus, a reliable and accurate method for the determination of Sb (III) is very necessary.

Several techniques including UV-visible spectrophotometry [10, 11], inductively coupled plasma mass spectrometry [12, 13], hydride generation atomic fluorescence spectrometry [14, 15], flame atomic absorption spectrometry [16–18], and electrothermal atomic absorption spectrometry [19–21] have been used for the determination of antimony species in various samples. Considering the poor sensitivities of flame atomic absorption spectrometry and UV-visible spectrophotometry, the more limited condition of hydride generation atomic fluorescence spectrometry, and the expensive price and analysis cost of inductively coupled plasma mass spectrometry, graphite furnace atomic absorption spectrometry (GFAAS) is an efficient alternative to determinate trace and ultratrace amounts of antimony. Besides the requirement of a relatively small injection volume, a partially eliminated matrix during the pyrolysis is another advantage of GFAAS. Despite the high sensitivity of GFAAS, it is still necessary to use separation techniques that allow preconcentration of antimony species, due to the complex matrix interferences and the low concentration of antimony species in water sample.

Many miniaturized techniques such as homogenous liquid-liquid microextraction [22, 23], solid phase extraction technology [24–26], cloud point extraction [2, 27], single drop microextraction [28–30], hollow-fiber liquid phase microextraction [17, 31], and dispersive liquid phase microextraction [32, 33] have been used as the processing methods of preconcentration. Also, these methods have been applied to preconcentration of antimony species. Compared with other methods, SDMM is a new and environmentally friendly sample pretreatment technology. It has the advantages of low cost, simple device, easy operation, very low amounts of organic solvent, and high enrichment efficiency. For its striking advantages, SDMM was selected as the preconcentration methods in our study. Moreover, considering the toxicity and flammability of organic solvents, the ionic liquid of 1-butyl-3-methylimidazolium hexafluorophosphate ([C_4_mim][PF_6_]) has been employed in SDME because of its environmental friendliness. As reported, ionic liquids have been used as novel solvents for the extraction of metal ions at room temperature [34–37]. Ionic liquid does not have detectable vapor pressure and it can avoid environmental and safety problems. Up till now, few analytical applications of SDME method based on [C_4_mim][PF_6_] for extraction and preconcentration of Sb (III) have been reported. Thus, further studies into the use of [C_4_mim][PF_6_] in SDME are important in order to improve existing methods.

In this study, a method for Sb (III) determination in water samples by SDME combined with GFAAS was proposed. BPHA and [C_4_mim][PF_6_] were employed as complexing agent and extraction solvent, respectively. The SDME system was fully characterized through optimizations of the relevant variables influencing the extraction of Sb (III).

## 2. Materials and Methods

### 2.1. Reagents

All the reagents were of analytical grade. The experiment water was double distilled deionized water purified by Millipore (Millipore, Bedford, MA, USA). Potassium antimony tartrate was from Tianjin Kemiou Chemical Reagent Co. Ltd., Tianjin, China. N-Benzoyl-N-phenylhydroxylamine (BPHA), sodium thiosulphate, hydrochloric acid, ammonia solutions oxine, dpy, dichloromethane (CH_2_Cl_2_), and trichloromethane (CHCl_3_) were bought from Aladdin (Aladdin Industrial Co., Shanghai, China). 1-Butyl-3-methylimidazolium hexafluorophosphate ([C_4_mim][PF_6_]) was bought from J&K (J&K Scientific Ltd., Beijing, China). The 1000 mg·L^−1^ Sb (III) stock solution was prepared by amounts of potassium antimony tartrate dissolved in double distilled deionized water. The pH of the Sb (III) stock solution was adjusted to 2.0 with 0.1 M hydrochloric acid and 0.1 M ammonia solution. 1×10^−3^ M BPHA was dissolved in methanol.

### 2.2. Instruments

Atomic absorption spectra were performed by a Perkin-Elmer 900T atomic absorption spectrometer (Perkin-Elmer, USA) equipped with transverse heated graphite atomizer, pyrolytic graphite coated tubes (Beijing, China), and an antimony hollow cathode lamp (Perkin-Elmer, USA) recommended by the manufacturer. In the whole operation, except for atomization mode, argon 99.99% was used as protective and purge gas, and the flow rate was 250 mL·min^−1^. The pH of all solutions was performed by pHB-3C (Shanghai Weiye Instrument Factory). And all the stir in this study was performed by 85-2A magnetic stirrer (Changzhou Aohua Instrument Co. Ltd.). Some instrumental parameters of GFAAS were as follows: the lamp current was 10 mA, the wavelength was 217.6 nm, the spectral bandpass was 0.7 nm, and the background correction was Zeeman.  Table 1 showed graphite furnace atomizer temperature-rising program.

### 2.3. Preparation of Samples

All the water samples were filtered through a 0.45 *μ*m pore size membrane filter to remove the suspended particulate matters and the pH of all the water samples was adjusted to 2.0 by using 0.1 M hydrochloric acid and 0.1 M ammonia solution. Water samples including bottled mineral water, river water (pH=6.2, Beijiang River, Shaoguan, China), and tap water were collected locally. Each of the treated water samples was preserved at 1.9 mL for the later determination.

### 2.4. Process of Single Drop Microextraction

The apparatus of SDME was the same as what we studied before [39]. 1.9 mL treated water sample or 1 *μ*g·L^−1^ Sb (III) standard solution and 100 *μ*L 1×10^−4^ M BPHA solution were added to a 5 mL vial. Microsyringe with 5 *μ*L of [C_4_mim][PF_6_] was positioned above the vial, and the needle was inserted through the septum. The tip of syringe needle was attached to a flared polytetrafluoroethylene tube. Then, the needle tip was immersed into the sample solution, and the microsyringe was pushed slowly in order to make the microdrop hang under the needle tip steadily. The time of the extraction was 6 min under the stirring rate of 600 rpm. After extraction, microdrop was inhaled into the microsyringe and injected into the graphite furnace atomic absorption spectrometer for analysis manually.

## 3. Results and Discussion

### 3.1. Chelating Agent and Extraction Solvent

**Figure 1:**
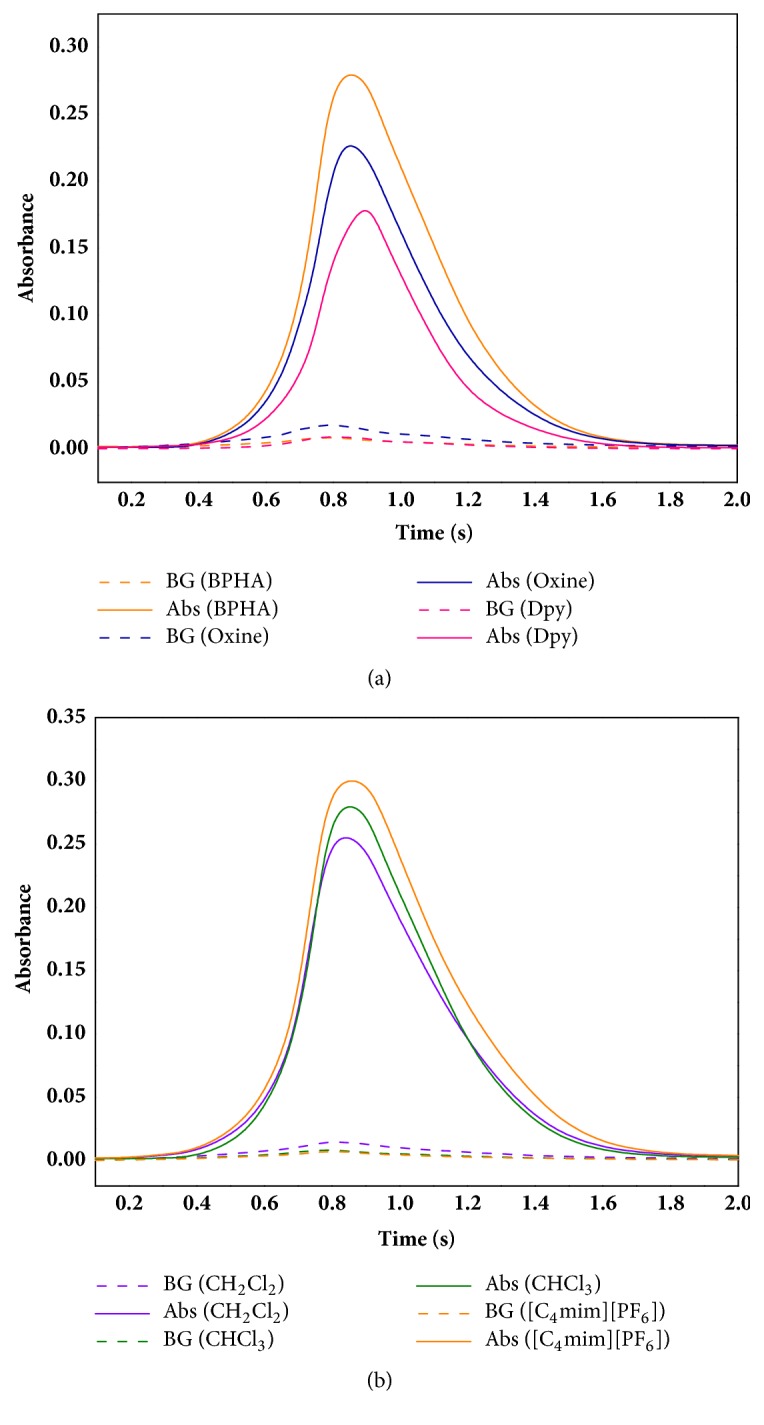
(a) The atomic absorbance of Sb (III) with different chelating agents such as BPHA, Oxine, and Dpy and their background absorbance. (b) The atomic absorbance of Sb (III) in different extraction solvents such as CH_2_Cl_2_, CHCl_3_, and [C_4_mim][PF_6_] and their background absorbance; BG: background absorbance without Sb (III); Abs: absorbance.

A chelating agent has a great influence on the extraction efficiency of Sb (III). Thus, a suitable chelating agent is very important.  Figure 1(a) showed the pattern of the atomic absorbance of Sb (III) with different chelating agents (BPHA, Oxine, and Dpy) and their background absorbance. It was found that the absorbance signal of Sb (III) with the chelating agent of BPHA was stronger than others. Although the absorbance signal of Sb (III) with Oxine was good, it had the stronger background interference. The absorbance signal of Sb (III) with Dpy had weaker signal compared with BPHA and Oxine. As a result, BPHA was selected as the chelating agent for SDME.

A suitable extraction solvent is also important for SDME. The density of the extraction solvent can be supposed to be higher than water so that it could keep the drop stable. CH_2_Cl_2_, CHCl_3_, and [C_4_mim][PF_6_] that were used in liquid-liquid extraction were evaluated as the extraction solvents. Each extraction solvent was dealt with via three different chelating agents, and then the method of SDME-GFAAS was applied to determine the amounts of Sb (III), and the results were shown in Table 2. Whatever the chelating agent was, [C_4_mim][PF_6_] always had the strongest signal.  Figure 1(b) described the atomic absorbance of Sb (III) in different extraction agents (CH_2_Cl_2_, CHCl_3_, and [C_4_mim][PF_6_]) with BPHA as the chelating agent and their background absorbance. Therefore, [C_4_mim][PF_6_] was selected as the extraction solvent for SDME.

### 3.2. Optimization of Single Drop Microextraction Conditions

#### 3.2.1. pH

**Figure 2:**
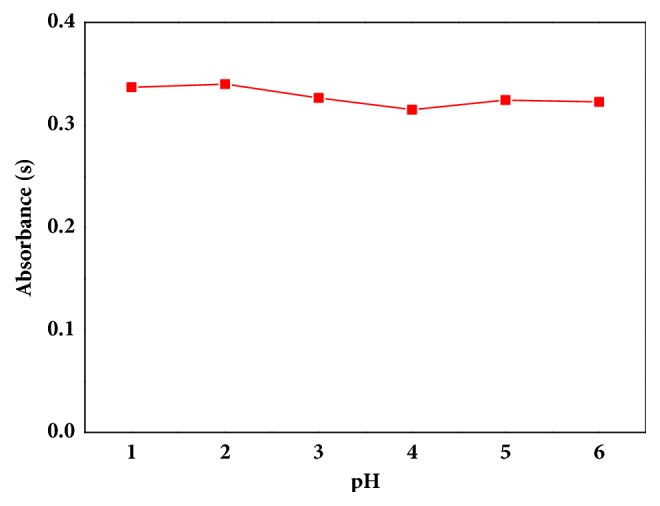
The atomic absorbance of Sb (III) in solutions with the pH value from 2.0 to 6.0.

The pH of the solution played an important role in the formation of metal chelate and the influence of the stability of chemicals. Also, it could affect the extraction of Sb (III) in the BPHA-[C_4_mim][PF_6_] system. The study of the pH was ranged from 1.0 to 6.0 and the results were shown in Figure 2. The absorbance signals of Sb (III) were high and less volatile in the range of 1.0-6.0. Considering that there may be interferences due to the competitive complexation reaction of other metal ions when the pH value was at a high level, pH=2.0 was chosen for the further study.

### 3.3. BPHA Concentration

**Figure 3:**
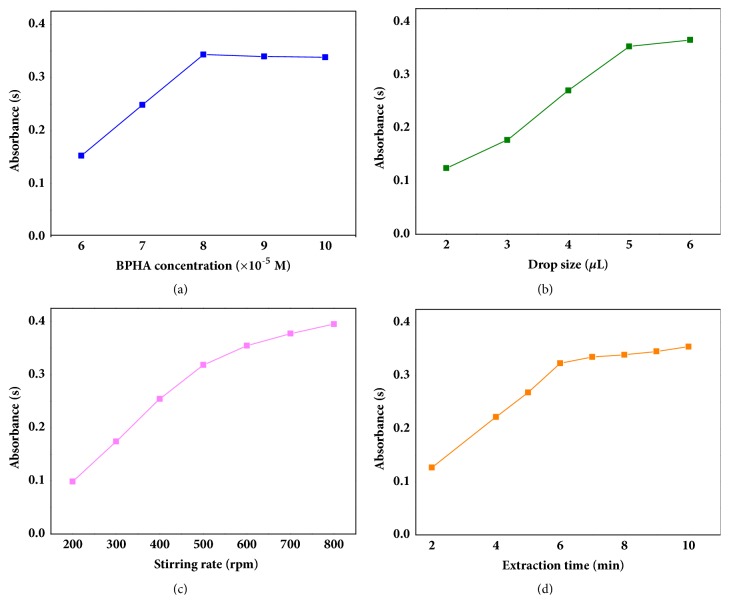
(a) The atomic absorbance of Sb (III) in solutions with the concentration of BPHA from 6×10^−5^ M to 10×10^−5^ M. (b) The atomic absorbance of Sb (III) in solutions with the drop size from 2.0 to 6.0 *μ*L. (c) The atomic absorbance of Sb (III) in solutions with the stirring rate from 200 to 800 rpm. (d) The atomic absorbance of Sb (III) in solutions with the extraction time from 2 to 10 min.

It was also necessary to find the minimal concentration of BPHA. The effect of BPHA concentration on the extraction efficiency of Sb (III) was investigated. The results were illustrated in Figure 3(a) and, as can be seen from it, the absorbance signal of Sb (III) increased with the BPHA concentration from 6×10^−5^ to 8×10^−5^ M and remained constant when the concentration of BPHA was above 8×10^−5^ M. To make the treatment easier, the value of 1×10^−4^ M was chosen for the further study.

### 3.4. Solvent Drop Size

The effect of drop size was shown in Figure 3(b), and it was found that the absorbance of Sb (III) increased with the increase of the drop size from 2.0 to 6.0 *μ*L. However, the drop size increasing usually resulted in the fall of the microdrop. In general, the stability of the microdrop depends on upward floating force, downward gravity, and adhesion forces [18]. In order to enhance the adhesion force of the microdrop, a flared polytetrafluoroethylene tube was attached to the tip of syringe needle. All these things were taken into account, and then 5.0 *μ*L was chosen as the drop size for extraction.

### 3.5. Stirring Rate

It was well known that the stirring rate could affect the extracting speed by changing the mass transfer in the sample solution. The effect of stirring rates on extraction efficiency was studied in the range of 200 to 800 rpm. The results in Figure 3(c) showed that the increasing stirring rate of the sample greatly improved the absorbance of Sb (III). However, the microdrop easily fell off the needle of the microsyringe when the stirring rate was above 600 rpm. Increasing stirring rate could also cause a reduction of [C_4_min][PF_6_] microdrop volume, because the dissolution of ionic liquid was enhancing. Thus, 600 rpm was selected as the best stirring rate in this study.

### 3.6. Extraction Time

The extraction efficiency depended on the length of the extraction time until the equilibrium was reached. Although the maximum sensitivity was achieved in equilibrium, complete equivalent was not necessary to obtain accurate analysis. Thus, the effect of extraction time on extraction efficiency had been studied from 2 to 10 min. The results were illustrated in Figure 3(d). There were a sharp increase from 2 to 6 min and a slow increase from 6 to 10 min. As the time went on, microdrop would fall into solutions. In order to avoid it, 6 min was selected as the extraction time which was enough for extracting Sb (III) for determination.

### 3.7. Optimization of Graphite Furnace Atomic Absorption Spectrometry

**Figure 4:**
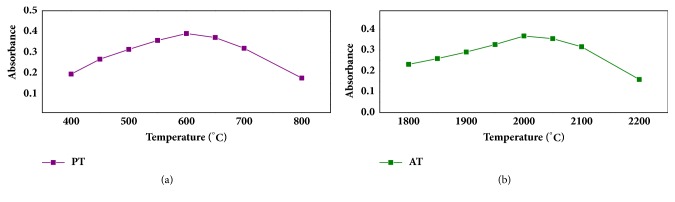
(a) The absorbance of Sb (III) with the pyrolysis temperature (PT) from 400°C to 800°C. (b) The absorbance of Sb (III) with the atomization temperature (AT) from 1800°C to 2200°C.

In order to reduce the chemical interference and the background signal, the work investigated the influence of pyrolysis temperature from 400°C to 800°C and atomization temperature from 1800°C to 2200°C. The 0.5 *μ*g·L^−1^ Sb (III) standard solutions were dealt with via the pretreatment of SDME and determined by GFAAS. As shown in Figure 4(b), background signals were stronger when the pyrolysis temperature was lower because of the excessive vaporization of BPHA and ionic liquid at atomization stage, and the strongest signal appeared at 2000°C. It was found in Figure 4(a) that the matrix was sufficiently eliminated and maximum absorbance was achieved at the pyrolysis temperature of 600°C; however the absorbance decreased with increasing of the pyrolysis temperature due to the loss of Sb at higher temperature. In addition, the time of atomization was 5 s. As the results showed in Figure 4, 2000°C was chosen as the atomization temperature and 600°C as the pyrolysis temperature.

### 3.8. Effect of Interferences

One of the interferences was other metal ions reacting with chelating agents and the other was coextraction. In order to validate the selectivity of Sb (III) in microextraction system, different amounts of ions were added to the 1.0 *μ*g·L^−1^ Sb (III) solutions, respectively. After determination, coexisting ions were considered to have interferences when the change of Sb (III) absorption value was more than 5%. As shown in the Results, the tolerance limit of coexisting ions including Na^+^, K^+^, Ca^2+^, Mg^2+^, Cl^−^, SO_4_^2−^, and PO_4_^3−^ was 2000 mg·L^−1^; of coexisting ions including Co^2+^, Cd^2+^, Cu^2+^, Mn^2+^, Zn^2+^, and Ag^+^ was 200 mg·L^−1^; of coexisting ions including Al^3+^, Cr^3+^, Fe^3+^, Ni^2+^, and Pb^2+^ was 50 mg·L^−1^.

### 3.9. Standard Analysis

As shown in the Results, the low limit of detection (LOD, 3*σ*) was 0.01 ng·mL^−1^, the relative standard deviation of 0.5 ng·mL^−1^ (n=6) was 4.2%, and the linear value ranged from 0.02 to 5 ng·mL^−1^. The regression equation was A=0.6832C+0.0034 (A represented the absorbance values and C represented the concentration of Sb (III) whose unit was ng·mL^−1^). Linear correlation coefficient was 0.999. A comparison of the main features of the proposed method with other reported methods in the literatures was shown in Table 3. This method was more effective for detecting Sb (III) with lower limit detection and had better precision than majority of the other reported methods.

### 3.10. Samples Analysis

A series of water samples were analyzed by the presented method. The results were shown in Table 4. The recoveries were in the ranges of 98-104% with the different standard of Sb (III) solutions spiked to the water samples. So, it demonstrated a good accuracy of this method.

## 4. Conclusions

The optimization method, combined with single drop microextraction using BPHA-[C_4_mim][PF_6_] system for separation of impurities, detected by GFAAS was developed to determinate trace Sb (III) in water samples. After a series of analysis of optimization conditions, an excellent accuracy, precision, and lower limit detection were obtained by this method. The relative standard deviation of the 0.5 *μ*g·L^−1^ Sb (III) was 4.2% (n=6). The detection limit (signal-to-noise ratio of 3) and the enrichment coefficient of Sb (III) were 0.01 *μ*g·L^−1^ and 112, respectively. What is more, the introduction of BPHA-[C_4_mim][PF_6_] system was not only ecofriendly comparing with traditional organic solution but also efficient for extraction. After the rapid extraction by SDME with the system of BPHA-[C_4_mim][PF_6_], the samples could be injected and detected directly. Thus, this method was a simple, effective, and environment-friendly way to determine the trace concentration of Sb (III) in water samples.

## Figures and Tables

**Table 1:** Graphite furnace atomizer temperature-rising program.

Steps	Temperature	Ramp time	Hold time	Argon flow rate
	(°C)	(s)	(s)	(mL·min^−1^)
Drying	120	10	15	250
Pyrolysis	600	5	20	250
Atomization	2000	0	5	0
Cleaning	2400	1	3	250

**Table 2:** The atomic absorbance of three chelating agents in three different extraction solvents.

Chelating agent	Extraction solvent		
	CH_2_Cl_2_	CHCl_3_	[C_4_mim][PF_6_]
Oxine	0.287	0.274	0.294
Dpy	0.158	0.143	0.176
BPHA	0.322	0.355	0.364

**Table 3:** Comparison of the proposed method with other methods for determination of antimony (III).

Method	Linear ranges	Limits of detection	Enrichment factor	Relative standard deviation	References
	(ng·mL^−1^)	(ng·mL^−1^)			
DLLME-ETAAS^a^	0.05-5	0.05	115	4.5%	[32]
CPE-ETAAS^b^	-	1.82	45	2.6%	[38]
VASEME-ETAAS^c^	0.4-8	0.09	53	5.4%	[20]
HFSLME-TAFFAAS^d^	5-200	0.8	160	7.8%	[17]
SDME-GFAAS	0.02-50	0.01	112	4.2%	This work

^a^Dispersive liquid-liquid microextraction-electrothermal atomic absorption spectrometry.

^b^Cloud point extraction- electrothermal atomic absorption spectrometry.

^c^Vortex-assisted surfactant-enhanced emulsification microextraction-electrothermal atomic absorption spectrometry.

^d^Hollow fiber supported liquid membrane extraction-thermospray flame furnace atomic absorption spectrometry.

**Table 4:** Determination of Sb (III) in water samples.

Samples	Added (*μ*g·L^−1^)	Found (*μ*g·L^−1^)	Recovery (%)
Bottle mineral water	0	< Limits of detection	-
	0.1	0.102 ± 0.01	102±1
	0.4	0.401 ± 0.008	100±2

River water	0	< Limits of detection	-
	0.1	0.104 ± 0.015	104±2
	0.4	0.407 ± 0.012	102±3

Tap water	0	< Limits of detection	-
	0.1	0.098 ± 0.011	98±1
	0.4	0.398 ± 0.009	99±2

## Data Availability

The [Graphite Furnace Atomic Absorption Spectrometry] data used to support the findings of this study are included within the article.
